# Health-related quality of life in the ENDEAVOR study: carfilzomib-dexamethasone vs bortezomib-dexamethasone in relapsed/refractory multiple myeloma

**DOI:** 10.1038/s41408-019-0181-0

**Published:** 2019-02-22

**Authors:** Heinz Ludwig, Philippe Moreau, Meletios A. Dimopoulos, Maria-Victoria Mateos, Martin Kaiser, Roman Hajek, Shibao Feng, Kim Cocks, Jaqueline Buchanan, Katja Weisel

**Affiliations:** 1 Wilhelminen Cancer Research Institute, Wilhelminenhospital, Vienna, Austria; 2 University of Nantes, Nantes, France; 3 National and Kapodistrian University of Athens School of Medicine, Athens, Greece; 4 University Hospital Salamanca, Salamanca, Spain; 5 The Institute of Cancer Research, London, UK; 6 University Hospital Ostrava and Faculty of Medicine, University of Ostrava, Ostrava, Czech Republic; 7 Amgen Inc, Thousand Oaks, CA USA; 8 KCStats Consultancy, Leeds, UK; 9 Universitatsklinikum Tubingen, Tubingen, Germany

## Abstract

We examined effects of carfilzomib-dexamethasone (Kd56) versus bortezomib-dexamethasone (Vd) on health-related quality of life (HR-QoL) in relapsed/refractory multiple myeloma (MM) patients from the ENDEAVOR study. HR-QoL was assessed by the European Organisation for Research and Treatment of Cancer QoL Questionnaire (QLQ-C30), MM-specific module (QLQ-MY20), and Functional Assessment of Cancer Therapy/Gynecologic Oncology Group-Neurotoxicity (FACT-GOG-Ntx) “Additional Concerns” neurotoxicity subscale. The QLQ-C30 Global Health Status (GHS)/QoL scale and seven prespecified subscales were compared between groups using mixed model for repeated measures. Of 929 randomized patients, 911 with ≥1 post-baseline assessment were included. Kd56 was associated with statistically significant improvements in GHS/QoL, fatigue, pain, side effects, and FACT/GOG-Ntx scores versus Vd, although mean differences did not meet thresholds for clinical significance. The Kd56 group had longer time to deterioration (TTD) in GHS/QoL (median 3.7 versus 2.8 months, *p* *=* 0.0046), physical function (5.6 versus 3.7 months, *p* *=* 0.0390), nausea/vomiting (17.6 versus 8.2 months, *p* *=* 0.0358), side effects (6.4 versus 3.7 months *p* *<* 0.0001), and FACT/GOG-Ntx (11.1 versus 5.5 months*, p* *=* 0.0004). Overall, Kd56 resulted in statistically but not clinically significant improvements in mean GHS/QoL scores versus Vd. Treatment with Kd56 versus Vd also significantly prolonged TTD in GHS/QoL, physical function, nausea/vomiting, side effects, and FACT/GOG-Ntx.

## Introduction

Although novel treatment options for multiple myeloma are associated with improvements in survival^1^, corresponding improvements in health-related quality of life (HR-QoL) have been limited^2^. Among the most distressing issues reported at diagnosis are reduced physical functioning, pain and fatigue, impairments in role functioning, and reduced overall HR-QoL^3^. As patients with multiple myeloma live longer with the disease and have increased access to a variety of new therapies, HR-QoL has grown in importance as an endpoint in clinical studies^2,4^.

Carfilzomib is an epoxyketone proteasome inhibitor that binds selectively and irreversibly to the proteasome. The combination of carfilzomib with dexamethasone (twice-weekly carfilzomib dose of 56 mg/m^2^; Kd56) is approved for the treatment of adult patients with relapsed or refractory multiple myeloma. The approval of Kd56 was based on the randomized, head-to-head, phase 3 ENDEAVOR study. ENDEAVOR showed a statistically significant prolongation of progression-free survival (primary endpoint) for patients with relapsed or refractory multiple myeloma who were treated with Kd56 compared with bortezomib and dexamethasone (Vd; median 18.7 versus 9.4 months; hazard ratio [HR] 0.53; 95% confidence interval [CI] 0.44 to 0.65; *p* *<* 0.0001)^5^. Patients treated with Kd56 versus Vd also had a statistically significant and clinically meaningful improvement in overall survival (median 47.6 months versus 40.0 months; HR 0.791; 95% CI 0.648 to 0.964; *p* *=* 0.010)^6^. HR-QoL was assessed as an exploratory endpoint in ENDEAVOR.

Here, we present full HR-QoL results from the ENDEAVOR study. Analyses of patient-reported outcomes (PROs) were prespecified in a separate statistical analysis plan. The primary PRO hypothesis was superiority of Kd56 over Vd for the Global Health Status/Quality of Life (GHS/QoL) scale. Further subscales were prespecified from the European Organisation for Research and Treatment (EORTC) Quality of Life Questionnaire-Core 30-item module (QLQ-C30) (fatigue, nausea/vomiting, pain, physical functioning, role functioning), the multiple myeloma specific quality of life 20-item module (QLQ-MY20) (disease symptoms, side effects of treatment), and the Functional Assessment of Cancer Therapy/Gynecologic Oncology Group-Neurotoxicity subscale (FACT/GOG-Ntx; neurotoxicity).

## Materials/subjects and methods

### Study design and participants

ENDEAVOR (NCT01568866) was a prospective, multicenter, open-label, randomized, phase 3 trial. Patients with relapsed or refractory multiple myeloma aged 18 years or older from 198 sites in North America, Europe, South America, and the Asia-Pacific region were recruited^5^. Full trial details have been published previously^5^.

Patients were randomized (1:1) using a stratified block randomization scheme, stratified by previous proteasome inhibitor therapy (yes versus no), previous lines of treatment (one versus two or three), International Staging System stage (I versus II or III), and planned route of bortezomib administration (intravenous versus subcutaneous) if randomly assigned to the Vd group. The Kd56 group received carfilzomib as a 30-min intravenous infusion (20 mg/m^2^ on days 1 and 2 of cycle 1; 56 mg/m^2^ given thereafter) on days 1, 2, 8, 9, 15, and 16 and dexamethasone (20 mg oral or intravenous infusion) on days 1, 2, 8, 9, 15, 16, 22, and 23 of a 28-day cycle. The Vd group received bortezomib (1.3 mg/m^2^) as an intravenous bolus or subcutaneous injection on days 1, 4, 8, and 11, and dexamethasone (20 mg oral or intravenous infusion) on days 1, 2, 4, 5, 8, 9, 11, and 12 of a 21-day cycle.

The study protocol was in accordance with the ethical standards of the institutional review boards or ethical committees of all participating institutions.

### HR-QoL assessments and endpoints

PROs were assessed with the EORTC QLQ-C30^7^, the disease-specific myeloma questionnaire (EORTC QLQ-MY20)^8^, and the neurotoxicity FACT/GOG-Ntx “Additional Concerns” questionnaire^9^. The EORTC QLQ-C30 and QLQ-MY20 were chosen as they have been extensively used and validated in patients with multiple myeloma^10–12^. They are quick to complete (less than 12 min on average together)^10^. The QLQ-C30 includes an overall GHS/QoL domain, five functional domains (physical, emotional, cognitive, social, and role functioning), and nine symptom domains (fatigue, nausea/vomiting, pain, dyspnea, insomnia, appetite loss, constipation, diarrhea, and financial difficulties). All domain scores range from 0 to 100. Higher scores on the overall GHS/QoL, functional, and symptom domains correspond to better HR-QoL, better functioning, or more severe symptoms, respectively, compared with lower scores. The QLQ-MY20 includes two functional domains (future perspective and body image) and two symptom domains (disease symptoms and side effects of treatment) with scores ranging from 0 to 100. Higher scores on the functional and symptom domain indicate better functioning and more symptoms, respectively, compared with lower scores. The 11-item neurotoxicity “Additional Concerns” subscale (Ntx subscale) is from the FACT/GOG-Ntx, which is a reliable and valid instrument for assessing the impact of neuropathy on HR-QoL in patients with ovarian cancer^9^ and has been used in trials evaluating multiple myeloma therapy^13,14^. The validity of the Ntx subscale in patients with relapsed myeloma was supported using data from another carfilzomib trial (PX-171-003-A1)^15^. The Ntx subscale is scored from zero to 44, with lower scores indicating more neurotoxic symptoms. Questionnaires were scored according to their respective scoring manuals.

PROs were completed by patients via electronic data capture (tablet). Patients completed the questionnaires prior to the start of drug administration on day 1 of cycle 1 (baseline), then every 28 days until disease progression, withdrawal of consent, or until they received another anticancer treatment. Due to the differing cycle lengths for the treatment groups, the timing of PRO assessments in relation to the cycle day varied. Every 12 weeks the PRO assessments coincided across the treatment groups on day 1 of a cycle. Post-treatment visit and further follow-up visits were collected but are not included in the analyses reported here in order to focus on the HR-QoL during treatment.

PRO hypotheses and analyses were prespecified in a statistical analysis plan. No adjustment for multiplicity was made because the PRO endpoints were defined as exploratory. The goal of the analysis was to determine whether Kd56 was superior to Vd with respect to the GHS/QoL score from the EORTC QLQ-C30. Further prespecified analyses were conducted with respect to QLQ-MY20 side effects and disease symptoms subscales, and the QLQ-C30 fatigue, nausea/vomiting, pain, physical functioning, and role functioning subscales.

The intention-to-treat population (all randomized patients) was used for the EORTC QLQ-C30, QLQ-MY20. In line with the analysis of adverse events, the Ntx subscale was analyzed using the safety population (all randomized patients receiving at least one dose of any study treatment and analyzed according to treatment received).

### Statistical analyses

Compliance was calculated using the proportion of randomized patients (intention-to-treat) with completed QLQ-C30 questionnaires and the proportion of patients expected to have an assessment (alive and on study treatment) with completed QLQ-C30 questionnaires. Missing data patterns were defined using tertiles to define early, middle, and late dropout groups based on patients’ last PRO assessment time. HR-QoL trajectories grouped by timing of dropout were plotted by treatment group to assess the trends by missing data pattern.

PRO subscales were compared between treatment groups using a restricted maximum likelihood-based mixed model for repeated measures (MMRM), assuming a constant treatment effect over time. The model included treatment and randomization stratification factors as fixed effects and random intercept and slope effects for patients. Baseline scores were accounted for using a constrained longitudinal data analysis approach^16,17^. Least squares means and 95% CIs are presented from the model. Two sensitivity analyses were planned for the GHS/QoL scale only to check robustness of the MMRM. One model included a term for dropout group (with patients split into early, middle, and late dropout groups based on tertiles specified using the data) to adjust for potential imbalance in dropout patterns between the two arms. The other model excluded timepoints past the point where more than 60% of the randomized population had missing data to check the robustness of the analysis results. An additional exploratory analysis was planned in order to check the impact of the different cycle length across treatment groups on the analysis of GHS/QoL. The model included the treatment-by-time interaction and analyzed the subset of visits where the HR-QoL assessment coincided with day 1 of a cycle for both of the treatment groups. To further assess any effect of different cycle lengths between treatment groups on the analysis of GHS/QoL, the primary MMRM model (without treatment-by-time interaction) was repeated in an exploratory analysis by including data only from the visits when patients in the Kd56 and Vd arms were at day 1 of their treatment cycle. A post-hoc sensitivity analysis assuming missing data was missing not at random was also carried out using a shared parameter model, jointly modeling the PRO data and time to last PRO assessment.

The MID for a PRO scale represents the smallest group-level difference in a PRO score that would be interpreted as clinically meaningful^18^. Clinical interpretation for the EORTC QLQ-C30 subscales was guided by prespecifying MIDs in the statistical analysis plan based on the evidence-based guidelines for the EORTC QLQ-C30^19^ and in line with others in the myeloma population (five points for the GHS/QoL)^19,20^. The EORTC QLQ-MY20 does not have any published MIDs. For the multi-item subscales, we proposed to use the standard error of measurement (SEM) as a proxy for the MID^21,22^. The MID for the FACT/GOG-Ntx score has yet to be determined but is estimated to be between 3.3 and 4.4 points^23^.

To explore individual patient changes in HR-QoL, HR-QoL responder analyses were conducted with patients classified as improved using a threshold of ≥5-point improvement in GHS/QoL score from baseline^22,24^. A sensitivity analysis using a more stringent threshold of a ≥15-point change was also conducted. The proportion of patients achieving a response on the GHS/QoL were compared between treatment groups at each timepoint where the PRO assessments coincided with day 1 of a cycle for both groups (every 12 weeks) using the Cochran-Mantel-Haenszel test stratified by randomization factors. Odds ratios and 95% CIs are reported. Patients with missing data were considered non-responders.

Time to deterioration in GHS/QoL was analyzed post hoc with the same response thresholds^24^. Cox proportional hazards models were used to account for the randomization stratification factors for all prespecified subscales. Patients with missing baseline assessments and/or no post-baseline assessments were censored at day 1. Patients without PRO subscales deterioration were censored at their last visits.

Longitudinal changes from baseline by treatment group for EORTC QLQ-C30 and QLQ-MY20 scores were explored post hoc using MMRM models; least squares mean estimates, 95% CIs, and *p* values were calculated. A two-sided 5% significance level was used. Clinical interpretation of changes from baseline for these scores was based on a comparison of the 95% CIs with guidelines by Cocks et al for interpreting longitudinal changes in QLQ-C30 scores^22,25^.

Change from baseline GHS/QoL scores for the subgroup of patients achieving a partial response (PR) or better was also explored post hoc using least squares mean estimates, 95% CIs, and *p* values from MMRM models. A two-sided 5% significance level was used.

### Role of the funding source

Amgen, Inc. was the study sponsor and played a role in the collection, analysis, and interpretation of data, in the writing of the report, and in the decision to submit the paper for publication.

### Data sharing statement

Qualified researchers may request data from Amgen clinical studies. Complete details are available at the following: http://www.amgen.com/datasharing

## Results

### Patient population

Between 20 June 2012 and 30 June 2014, a total of 929 patients were randomized to Kd56 (*n* = 464) or Vd (*n* = 465)^5^. The majority (79%) of patients in the Vd group received subcutaneous bortezomib throughout the entire treatment period; the remaining patients in the Vd group received intravenous bortezomib at least once during the treatment period^5^.

**Fig. 1:**
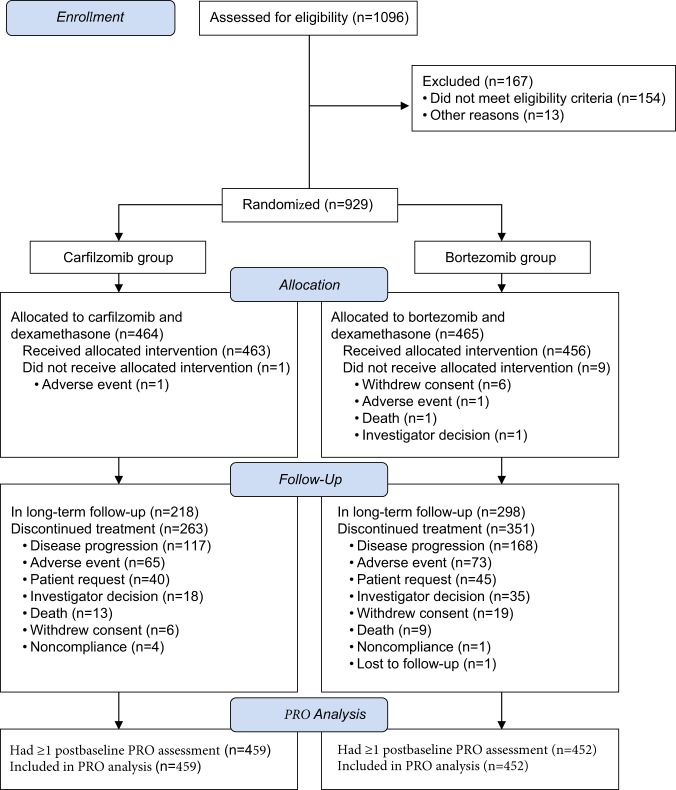
Subject disposition (CONSORT diagram). *PRO* patient-reported outcome

Among the randomly assigned patients, a total of 911 had at least one post-baseline PRO assessment before end-of-treatment and were included in the PRO analyses (Fig. 1; Kd56, *n* = 459; Vd, *n* = 452). Baseline characteristics were generally similar between treatment groups^5^. Table 1 presents baseline summary scores for the 15 subscales of the QLQ-C30, the four subscales of the QLQ-MY20, and the FACT/GOG-Ntx “Additional Concerns”. The baseline scores for the QLQ-C30 and QLQ-MY20 were similar between the treatment groups for all subscales except for insomnia. A baseline mean difference larger than the MID was observed for insomnia, with patients in the Kd56 group reporting more problems than those in the Vd group (27.2 versus 20.9 respectively). The baseline mean scores for the FACT/GOG-Ntx “Additional Concerns” were the same for each group (37.0).

**Table 1:** Baseline patient characteristics and PROs

	Kd56 (*n* = 464)	Vd (*n* = 465)
Age (years)^a^		
Median (range)	65 (35–89)	65 (30–88)
<65	223 (48%)	210 (45%)
65–74	164 (35%)	189 (41%)
≥75	77 (17%)	66 (14%)
Sex^a^		
Male	240 (52%)	229 (49%)
Female	224 (48%)	236 (51%)
ECOG performance status^a^		
0	221 (48%)	232 (50%)
1	211 (45%)	203 (44%)
2	32 (7%)	30 (6%)
Geographical region^a^		
Eastern Europe	135 (29%)	121 (26%)
Western Europe	182 (39%)	169 (36%)
North America	35 (8%)	49 (11%)
South America	10 (2%)	15 (3%)
Asia Pacific	102 (22%)	111 (24%)
History of peripheral neuropathy^a^		
No	249 (54%)	221 (48%)
Yes	215 (46%)	244 (52%)
Ongoing peripheral neuropathy at screening^a^		
Grade 1	133 (29%)	159 (34%)
Grade 2	10 (2%)	10 (2%)
Previous proteasome inhibitor treatment^a,b^		
Bortezomib	250 (54%)	252 (54%)
Carfilzomib	2 (<1%)	1 (<1%)
None	212 (46%)	212 (46%)
Previous immunomodulatory agent treatment^a^		
Lenalidomide	177 (38%)	177 (38%)
Thalidomide	211 (45%)	247 (53%)
**EORTC QLQ-C30 functional domain scores, mean (SD)**		
QLQ-C30 Global Health Status/QoL	61.5 (21.3)	63.7 (21.7)
Physical functioning	73.8 (22.0)	74.0 (22.0)
Role functioning	73.7 (28.5)	73.6 (28.6)
Emotional functioning	76.3 (20.1)	77.3 (19.4)
Cognitive functioning	83.7 (18.6)	84.7 (18.5)
Social functioning	77.4 (26.0)	79.5 (23.9)
**EORTC QLQ-C30 symptom domain scores, mean (SD)**		
Fatigue	33.6 (23.3)	34.5 (24.0)
Nausea/vomiting	4.9 (11.3)	5.2 (12.7)
Pain	33.1 (28.5)	31.2 (27.4)
Dyspnea	19.2 (23.7)	16.8 (23.4)
Insomnia	27.2 (29.3)	20.9 (26.6)
Appetite loss	15.7 (25.6)	13.4 (23.1)
Constipation	12.8 (22.5)	12.8 (22.1)
Diarrhea	7.2 (17.7)	7.3 (18.1)
Financial difficulties	17.9 (27.7)	15.9 (25.2)
**QLQ-MY20 scores, mean (SD)**		
Disease symptoms	27.8 (20.5)	25.1 (20.6)
Side effects	16.8 (13.7)	16.8 (13.3)
Future perspective	58.1 (24.0)	62.4 (23.8)
Body image	79.2 (27.5)	82.3 (26.6)
**FACT-GOG/Ntx score, mean (SD)**	37.0 (6.0)	37.0 (6.3)

*ECOG* Eastern Cooperative Oncology Group, *EORTC* European Organisation for Research and Treatment of Cancer, *Kd56* carfilzomib (56 mg/m^2^) and dexamethasone, *PRO* patient-reported outcome, *QLQ-C30* EORTC core Quality of Life Questionnaire, *QLQ-MY20* EORTC Quality of Life Questionnaire myeloma-specific, *QoL* quality of life, *SD* standard deviation, *Vd* bortezomib and dexamethasone

^a^Presented in Dimopoulos MA, et al.^5,6^

^b^Defined as patients who achieved at least a partial response and had at least 6 months since last proteasome inhibitor treatment; all patients who had received previous carfilzomib and all except one patient who had received previous bortezomib met this definition for previous proteasome inhibitor therapy^5^

### Compliance

Compliance was similar across the PRO instruments. Table 2 is based on returned QLQ-C30 questionnaires, and similar rates were observed for the calculated GHS/QoL scores. The extent of missing data was slightly higher in the Vd group compared with the Kd56 group (16 versus 12%, respectively). Baseline compliance was similar between the two treatment arms. Compliance was high as a proportion of the number of patients expected to provide a questionnaire at each timepoint (patients who were alive and on-study), ranging from 73 to 94%. However, compliance in the Kd56 group was consistently higher than in the Vd group. As a proportion of the patients randomized, less than 40% of patients remained in the study after week 40 in the Kd56 group and week 24 in the Vd group. The median duration on study treatment was 40 weeks and 27 weeks for patients randomized to Kd56 and Vd, respectively.

**Table 2:** Extent of missing QLQ-C30 questionnaires (intention-to-treat)

	Kd56 (*n* = 464)	Vd (*n* = 465)	Kd56 (*n* = 464)	Vd (*n* = 465)
	Number (%) of patients with QLQ-C30 questionnaire completed out of number of randomized patients		Number (%) of patients with QLQ-C30 questionnaire completed out of number of expected patients	
Baseline	407/464 (87.7)	392/465 (84.3)	407/464 (87.7)	392/465 (84.3)
Week 12	383/464 (82.5)	336/465 (72.3)	383/408 (93.9)	336/388 (86.6)
Week 24	298/464 (64.2)	222/465 (47.7)	298/343 (86.9)	222/254 (87.4)
Week 36	235/464 (50.6)	142/465 (30.5)	235/258 (91.1)	142/162 (87.7)
Week 48	137/464 (29.5)	73/465 (15.7)	137/159 (86.2)	73/80 (91.3)
Week 60	73/464 (15.7)	28/465 (6.0)	73/82 (89.0)	28/37 (75.7)
Week 72	41/464 (8.8)	11/465 (2.4)	41/44 (93.2)	11/12 (91.7)
Post-treatment visit^*^	176/464 (37.9)	240/465 (51.6)	176/264 (66.7)	240/360 (66.7)

*Kd56* carfilzomib (56 mg/m^2^) and dexamethasone, *QLQ-C30* Quality of Life Questionnaire-Core 30-item module, *Vd* bortezomib and dexamethasone

*Post-treatment visit (or end-of-treatment visit) approximately 30 days after discontinuation of all study drugs or before start of subsequent treatment (whichever occurred first)

### Missing data patterns

The early dropout group was defined by dropout before week 24, the middle group between week 24 and week 40, and the late group from week 44 to week 72. The Kd56 group had a lower proportion of patients in the early dropout group than did the Vd group (22 versus 40%); conversely, the Kd56 group had a higher proportion in the late dropout group than did the Vd group (42 versus 25%).

Graphs of GHS/QoL scores over time stratified by dropout groups demonstrate very similar trends between the treatment groups (Supplementary Figure S1). The early dropout group started at a lower baseline HR-QoL, and the majority of patients who dropped out in this group had declining scores prior to dropout. In contrast, the middle and late dropout groups started at a similar, higher baseline. The middle and late dropout groups appear to be stable or improving prior to dropout.

### QLQ-MY20 MID

Internal consistency of the QLQ-MY20 multi-item subscales was good (Cronbach’s alpha > 0.7). The SEM was 9 for disease symptoms, 10 for future perspective, and 7 for side effects of treatment. The SEMs were very similar to those found in previous studies, including the ASPIRE carfilzomib study^21,22^.

### Treatment group differences

#### QLQ-C30 GHS/QoL scores

**Fig. 2:**
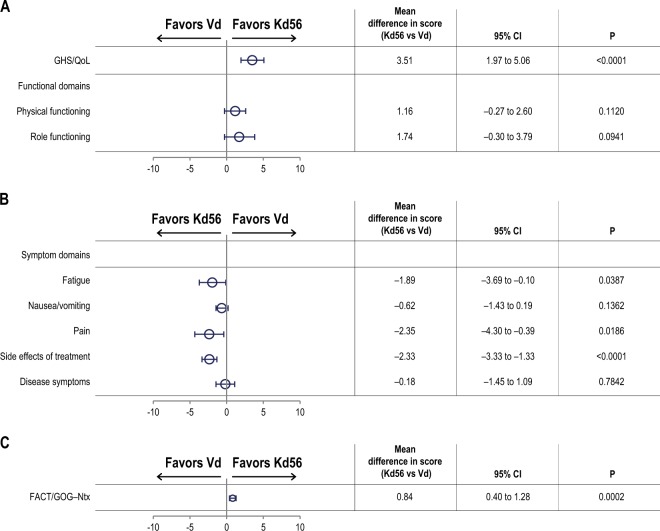
Adjusted least squares mean treatment difference in QLQ-C30 and QLQ-MY20 scores, and functional assessment of the FACT/GOG-Ntx subscale. **a** GHS/QoL and functional domains from the QLQ-C30. *: **b** Symptom domains from the QLQ-C30 and QLQ-MY20. †: **c** FACT/GOG-Ntx. ‡: Horizontal bars indicate 95% confidence intervals. The analysis was performed based on a linear mixed-effects model. The model includes the fixed, categorical effects of treatment, the randomization stratification factors—prior proteasome inhibitor treatment, lines of prior treatment, International Staging System stage and choice of route of bortezomib administration, and random effects of subject intercept and coefficient on time. The least squares mean estimates are the overall estimates under the assumption that the treatment effect is the same across visits. Prespecified, between-group MIDs for QLQ-C30 subscales were 5 for GHS/QoL, 6 for physical functioning, 7 for role functioning, 6 for fatigue, 4 for nausea/vomiting, and 7 for pain^19^. Between-group MIDs for QLQ-MY20 scales using the SEM as a proxy were 9 for disease symptoms and 7 for side effects of treatment. The MID for the FACT/GOG-Ntx score is estimated to be between 3.3 and 4.4 points^23^. *: Subscales were scored as directed by the EORTC scoring manual where higher scores indicate better HR-QoL and better functioning. †: Subscales were scored as directed by the EORTC scoring manual where higher scores indicate more severe symptoms (QLQ-C30) or more symptoms (QLQ-MY20). ‡: The Ntx subscale is scored from zero to 44, with lower scores indicating more neurotoxic symptoms. *EORTC:* European Organisation of Research and Treatment of Cancer; *FACT/GOG-Ntx:* Functional Assessment of Cancer Therapy/Gynecologic Oncology Group-Neurotoxicity subscale; *GHS/QoL*: Global Health Status/Quality of Life domain; *HR-QoL:* health-related quality of life; *Kd56:* carfilzomib (56 mg/m^2^) and dexamethasone; *MID*: minimum important difference; *QLQ-C30:* Quality of Life Questionnaire-Core 30-item module; *QLQ-MY20:* Quality of Life Questionnaire-multiple myeloma specific 20-item module; *SEM*: standard error of the mean; *Vd:* bortezomib and dexamethasone

**Fig. 3:**
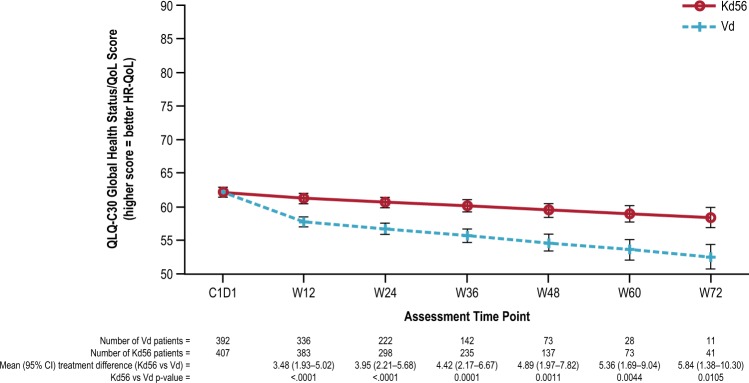
Least square mean estimates and standard errors by treatment group (for the subset of visits with assessments at day 1 of a cycle) in QLQ-C30 Global Health Status/QoL. Note: analysis was performed based on a linear mixed-effects model. The model included the fixed, categorical effects of treatment (all baseline responses are modeled with a dummy treatment), the randomization stratification factors—prior proteasome inhibitor treatment (prior carfilzomib or bortezomib versus no prior carfilzomib or bortezomib treatment), lines of prior treatment (1 versus 2 or 3), International Staging System (1 versus 2 or 3) and choice of route of bortezomib administration (intravenous or subcutaneous), and random effects of patient intercept and coefficient on time and treatment-by-time interaction. The prespecified between group MID for GHS/QoL was 5. *C1D1* cycle 1 day 1, *HR-QoL* health-related quality of life, *Kd56* carfilzomib (56 mg/m^2^) and dexamethasone, *QLQ-C30* Quality of Life Questionnaire-Core 30-item module, *MID* minimum important difference, *QoL* quality of life, *Vd* bortezomib and dexamethasone, *W* week

GHS/QoL mean treatment differences, least squares mean scores, and descriptive mean scores are shown in Figs. 2–3 and Supplementary Figure S2. Using the primary MMRM model, Kd56 was associated with statistically significantly higher GHS/QoL scores compared with Vd (Fig. 2; *p* *<* 0.0001). However, the overall treatment difference point estimate of 3.5 (95% CI 2.0 to 5.1) did not reach the predefined MID. When including the treatment-by-time interaction (*p* *=* 0.28) to estimate the treatment difference at timepoints where HR-QoL assessments coincided with day 1 of a cycle, the difference in point estimates increased over time (Fig. 3). The descriptive means by treatment group at each visit are shown in Supplementary Figure S2. Restricting the primary MMRM model to include data only from visits when patients in both treatment groups were at day 1 of their treatment cycle resulted in an overall treatment difference point estimate of 3.2 (95% CI, 1.2 to 5.2; *p* = 0.0019).

Results from the three sensitivity analyses confirmed findings from the MMRM analysis. The first analysis accounting for dropout pattern (early, middle, or late) showed a statistically significant result (2.6, 95% CI 1.1 to 4.1, *p* *=* 0.0009 which did not meet the predefined MID). The second analysis excluded data collected when more than 60% of randomized patients dropped out (i.e., excluding visits after week 36) and demonstrated that the results are robust despite the smaller patient numbers at later visits, with a statistically significant treatment difference (Kd56–Vd difference of 3.3 [95% CI 1.7 to 4.9], *p* *<* 0.0001). The third analysis, the shared parameter model, also supports the conclusions from the MMRM of a statistically significant benefit of Kd56 versus Vd on GHS/QoL scores. The overall estimated treatment effect was 4.4 (95% CI 2.8 to 6.0, *p* *<* 0.0001). In this model, correlation between the time to last PRO assessment and the random effects slope was not significant, indicating the rate at which PRO scores change over time does not appear to be associated with dropout.

#### A priori subscales of the QLQ-C30, QLQ-MY20 and FACT/GOG-Ntx

There were statistically significant benefits in favor of the Kd56 group for fatigue (*p* *=* 0.04), pain (*p* *=* 0.02), side effects (*p* *<* 0.0001), and Ntx subscales (*p* *=* 0.0002), although the difference between groups did not reach the MID (Fig. 2)^19^.

#### HR-QoL responder analysis

**Fig. 4:**
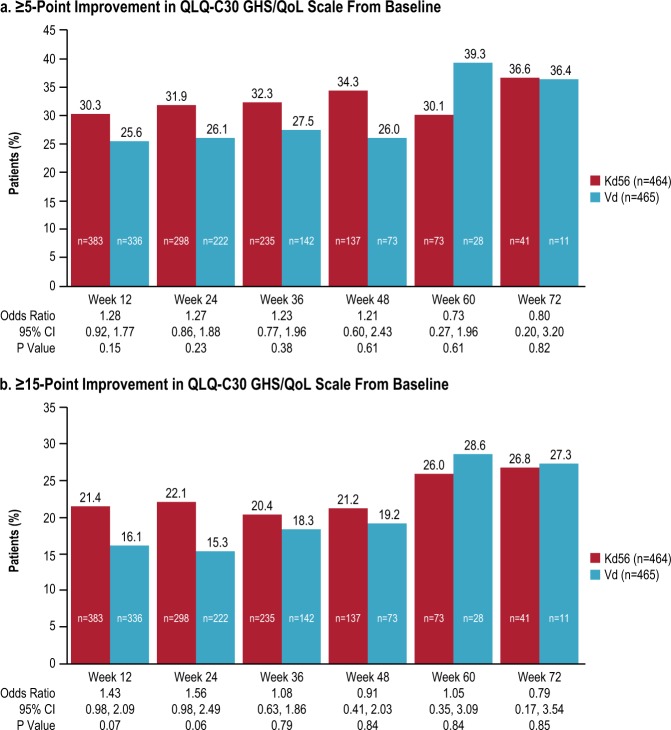
Percentage of PRO responders for QLQ-C30 GHS/QoL. **a** ≥5-point improvement in QLQ-C30 GHS/QoL scale from baseline. **b** ≥15-point improvement in QLQ-C30 GHS/QoL scale from baseline. Odds ratios are estimated using the Cochran-Mantel-Haenszel method stratified by the following randomization stratification factors: prior proteasome inhibitor treatment (yes versus no), lines of prior treatment (1 versus 2 or 3 lines), International Staging System stage (1 versus 2 or 3), choice of route of bortezomib administration (intravenous versus subcutaneous). *GHS/QoL* Global Health Status/Quality of Life, *Kd56* carfilzomib (56 mg/m^2^) and dexamethasone, *QLQ-C30* Quality of Life Questionnaire-Core 30-item module, *Vd* bortezomib and dexamethasone

The proportion of patients reaching at least 5-point improvement in the GHS/QoL scale was higher in the Kd56 group up to week 48, although the difference between the groups did not reach statistical significance (Fig. 4a). For the sensitivity analysis (at least 15-point improvement), the proportion of patients who improved at week 12 was 21.4 and 16.1% in the Kd56 and Vd groups, respectively (*p* *=* 0.0658), and at week 24 was 22.1 and 15.3% (*p* *=* 0.0584; Fig. 4b). At weeks 36, 48, 60, and 72 the difference in proportions were smaller and not statistically significantly different.

Patients in the Kd56 group also experienced a longer time to deterioration in GHS/QoL compared with those in the Vd group (HR from Cox model 0.77; 95% CI 0.65 to 0.92; *p* *=* 0.0046), with a median time to deterioration (≥15-point reduction) of 3.7 versus 2.8 months, respectively. Median time to deterioration (10 points) was also greater for the Kd56 group versus Vd group on physical function (5.6 versus 3.7 months; HR 0.82; 95% CI 0.68 to 0.99; *p* *=* 0.0390), nausea/vomiting (17.6 versus 8.2 months; HR 0.78; 95% CI 0.62 to 0.98; *p* *=* 0.0358) and side effects (6.4 versus 3.7 months; HR 0.65; 95% CI 0.54 to 0.78; *p* *<* 0.0001). Median time to deterioration (5-points) was greater for Kd56 versus Vd group on FACT/GOG-Ntx (11.1 versus 5.5 months; HR 0.69; 95% CI 0.56 to 0.85; *p* *=* 0.0004). No differences in time to deterioration were observed for the other prespecified subscales. FACT-GOG/Ntx data were not collected beyond treatment/progression.

#### Longitudinal changes by treatment group for EORTC QLQ-C30 and QLQ-MY20

**Fig. 5:**
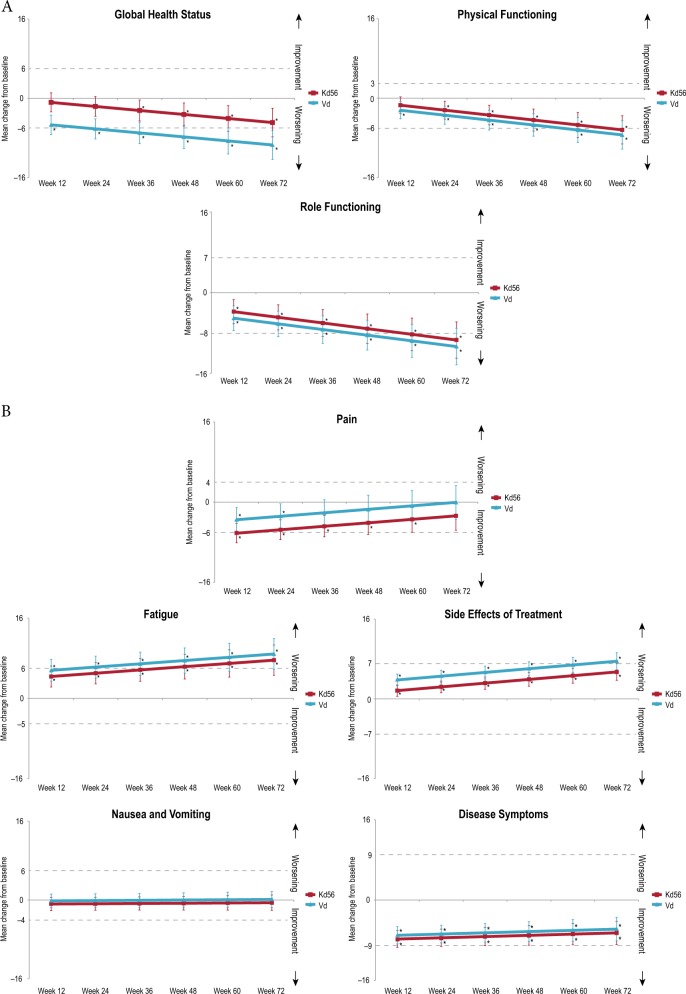
Estimated least squares mean change from baseline for the QLQ-C30 and QLQ-MY20 scores. **a** GHS/QoL and functional domains from the QLQ-C30. †: **b** Symptom domains from the QLQ-C30 and QLQ-MY20 ‡. Vertical bars indicate 95% confidence intervals of the mean. *p *<* 0.05 change from baseline. †: Subscales were scored as directed by the EORTC scoring manual where higher scores indicate better HR-QoL and better functioning. ‡: Subscales were scored as directed by the EORTC scoring manual where higher scores indicate more severe symptoms (QLQ-C30) or more symptoms (QLQ-MY20). Clinically meaningful changes for improvements and worsening are indicated by dotted horizontal lines for the QLQ-C30 scales^25^. SEM (±1 SEM) has been used in absence of anchor-based minimally important change for the QLQ-MY20^22^. *EORTC:* European Organisation of Research and Treatment of Cancer; *HR-QoL* health-related quality of life, *Kd56* carfilzomib (56 mg/m^2^) and dexamethasone, *QLQ-C30* Quality of Life Questionnaire-Core 30-item module, *QLQ-MY20* Quality of Life Questionnaire-multiple myeloma specific 20-item module, *Vd* bortezomib and dexamethasone

Figure 5 shows the change from baseline for each treatment group. The Vd group experienced statistically significant and clinically meaningful worsening in GHS/QoL and fatigue from week 24, role functioning from week 48, physical functioning from week 60, and side effects of treatment at week 72. The Kd56 group showed statistically significant and clinically meaningful worsening in fatigue from week 48, role functioning from week 60 and physical functioning at week 72; an early improvement in pain (week 12) was also observed. Changes in other subscales did not reach clinical significance.

#### HR-QoL for patients achieving a PR or better

**Fig. 6:**
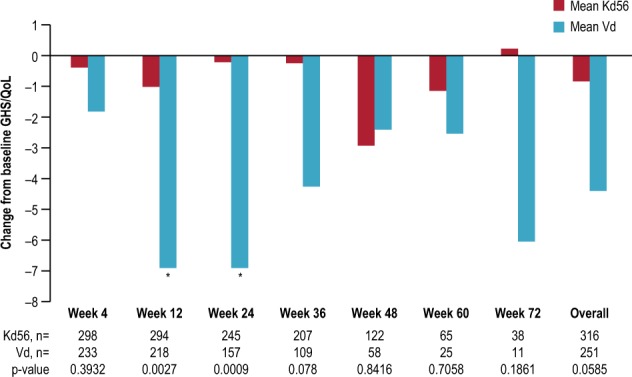
Change from baseline GHS/QoL domain for patients with a partial response or better. Positive change indicates improved GHS/QoL. The clinically meaningful threshold for improvements and worsening in the GHS/QoL domain was ±6^25^. *p < 0.05 between-group differences. *GHS/QoL* Global Health Status/Quality of Life, *Kd56* carfilzomib, lenalidomide, and dexamethasone, *Vd* bortezomib and dexamethasone

Figure 6 shows the change from baseline GHS/QoL for patients with a PR or better at each cycle and overall. A total of 316 patients treated with Kd56 and 251 patients treated with Vd achieved a PR or better and had baseline GHS/QoL assessment and at least one post-baseline assessment. The Kd56 responders (>PR) showed less deterioration in GHS/QoL scores from baseline compared with Vd responders across most cycles. Differences between the groups were statistically significant and clinically relevant at week 12 and 24. For Kd56 responders, the proportion of patients with maintained or improved GHS/QoL scores from baseline ranged from 55 to 74% over time; for Kd56 non-responders, this range was 43 to 100% across assessment timepoints. For Vd responders, the proportion of patients with maintained or improved GHS/QoL scores from baseline ranged from 47 to 58%; for the Vd non-responders this range was 0–100% across assessment timepoints.

## Discussion

Relapsed or refractory multiple myeloma can be experienced as either an acute or chronic condition^22,26–28^. One of the major goals of multiple myeloma treatment is to improve or maintain HR-QoL. For many chronic, disabling conditions, the intention of drug therapy is not necessarily to cure but to ameliorate symptoms, facilitate functioning, or improve HR-QoL^29^. The functional impairment and reduced independence associated with symptoms and adverse events, in conjunction with the burden of living with a terminal illness, can have a profound impact on overall HR-QoL^22^. Assessing change in overall health over time (i.e., GHS/QoL) in the context of a clinical trial provides valuable patient perspective on the combined impact of symptomatic and functional impairment. ENDEAVOR was the first head-to-head phase 3 study comparing two proteasome inhibitors for the treatment of patients with relapsed or refractory multiple myeloma and it included PROs as prespecified exploratory endpoints. The results of the primary PRO analysis demonstrated that patients treated with Kd56 had statistically superior GHS/QoL scores compared with patients treated with Vd, but these did not reach the prespecified MID (5 points). A declining trend in scores was observed in both treatment groups. Three sensitivity analyses were performed which confirmed the results of the primary analysis.

Statistically significant differences were also observed for three of the prespecified domains (fatigue, pain, and side effects of treatment), with the Kd56 group having lower symptom scores compared with the Vd group. These differences were, however, small and did not reach the prespecified MIDs, and were therefore unlikely to be clinically relevant. There were no differences between Kd56 and Vd for the remaining prespecified domains of nausea/vomiting, physical functioning, role functioning, side effects, and disease symptoms.

The delay in time to deterioration was significantly longer for Kd56 versus Vd for global HR-QoL, physical, nausea/vomiting, and side effects. The doubling of progression-free survival in the ENDEAVOR trial is associated with a prolonged period of time before deterioration of HR-QoL in the Kd56 versus Vd group; this is particularly relevant given that patients’ HR-QoL steadily degrades as the disease progresses and patients relapse and develop resistance to therapy^30^.

In the ENDEAVOR study, Kd56 demonstrated superiority over Vd with significantly lower rates of grade ≥ 2 peripheral neuropathy, a prespecified secondary endpoint (6% versus 32%; *p* *<* 0.0001)^5^. Treatment discontinuation due to peripheral neuropathy occurred in zero of 463 patients in the carfilzomib group, compared with 10 (2%) of 456 patients in the bortezomib group. We expected this to translate into patient-reported differences on the neurotoxicity subscale of the FACT/GOG-Ntx. While subjects treated with Kd56 had on average higher FACT/GOG-Ntx scores with an overall difference between Kd56 and Vd groups of 0.84 (indicating lower neurotoxicity for Kd56 versus Vd), this is unlikely to be a clinically relevant difference, as the magnitude of difference is small. However, the MID on this scale is estimated rather than established^23^. It is possible that missing data contributed to the lack of clinically meaningful difference. Post-hoc exploratory analyses indicated that the treatment difference varied over time and the time to deterioration of FACT/GOG-Ntx scores was significantly longer for the Kd56 patients (11.2 months versus 5.6 months in the Vd patients).

Our findings expand upon previous studies related to HR-QoL in multiple myeloma patients. The phase 3 FIRST trial evaluated the impact of continuous lenalidomide and low-dose dexamethasone compared with melphalan, prednisone, and thalidomide on HR-QoL^31^. In a recent analysis of patients with relapsed or refractory multiple myeloma from the ASPIRE trial, improved HR-QoL was associated with carfilzomib treatment^22^. Patients who were given carfilzomib, lenalidomide, and dexamethasone (KRd) had higher GHS/QoL scores over 18 treatment cycles compared with patients given lenalidomide and dexamethasone (Rd) (two-sided *p* *=* 0.0001). In addition, a higher proportion of KRd versus Rd patients met the GHS/QoL responder definition (≥5-point improvement)^22^. Our analysis provides the first evidence of longer time to deterioration in HR-QoL with Kd56 compared with the standard Vd regimen.

This study has several limitations. This was an open-label trial, and patients were aware of their treatment allocation prior to completing their baseline assessment and during all subsequent assessments. Despite the open-label design, both study arms had similar baseline completion rates. Mean baseline scores for the Kd56 group were slightly lower than those for the Vd group at baseline, although these differences were generally not clinically meaningful (smaller than the MID) and the observed difference in insomnia may have been due to chance. In addition, there was a tendency towards higher attrition in the Vd group. However, Bell and colleagues demonstrated that differential attrition does not necessarily result in bias, and recommend an analysis strategy such as used in this study to check the robustness of the analysis results^34^. The congruency of the primary and sensitivity analyses suggest that the finding of higher GHS/QoL scores in the Kd56 group compared with the Vd group is robust. The randomized treatments had different cycle lengths; therefore, some assessments may have penalized the Vd group by measuring HR-QoL mid-cycle compared to day 1 in the Kd56 group. However, analyses including only the assessments with both treatment groups at day 1 indicate that this bias was very small. In addition, the Kd56 group was treated for a longer period of time than the Vd group, allowing for more adverse event and other data collection in the Kd56 group. Finally, intravenous and twice-weekly bortezomib administration have been associated with higher rates of peripheral neuropathy compared with subcutaneous or once-weekly bortezomib administration^32,33^. Although 79% of the patients in the Vd arm in ENDEAVOR received bortezomib subcutaneously, they started treatment with a twice-a-week schedule^5^. The twice-weekly schedule along with intravenous administration of bortezomib have become less common as subcutaneous bortezomib with or without once-weekly dosing have become increasingly used.

The goals of multiple myeloma therapy include disease control and, ultimately, prolonged survival and maximized well-being^22^. However, extending survival should lead to assurance that HR-QoL is also improved, or at least maintained for longer. The ENDEAVOR study is the first head-to-head phase 3 trial comparing two proteasome inhibitors in patients with relapsed or refractory multiple myeloma. The ENDEAVOR trial showed significant superiority of Kd56 versus Vd in progression-free survival, overall survival, and overall response rates. Our results demonstrate a declining trend in mean GHS/QoL scores was observed in both study arms. The QLQ-C30 GHS/QoL subscale scores were higher in the Kd56 group than in the Vd group, with statistically, but not clinically, significant differences between the groups. Longer TTD for Kd56 versus Vd was observed in GHS/QoL, physical function, nausea/vomiting, side effects and FACT/GOG-Ntx. Overall, these results suggest that Kd56 should be considered for patients with relapsed or refractory multiple myeloma receiving a proteasome inhibitor.

## Supplementary information

Supplementary Information.
