# Supplementary material for: Health-related quality of life in the ENDEAVOR study: carfilzomib-dexamethasone vs bortezomib-dexamethasone in relapsed/refractory multiple myeloma
Source: Blood Cancer J. 2019 Feb 22;9(3):23. doi: 10.1038/s41408-019-0181-0 (PMC6386751; doi:10.1038/s41408-019-0181-0)

**Supplementary Information**

**Fig. S1 Mean QLQ Global Health Status/QoL score over time stratified by dropout pattern. a** Kd56 group. **b** Vd group. *C1D1*: cycle 1 day 1; *EORTC:* European Organisation for Research and Treatment of Cancer; HRQL: health-related quality of life; *Kd56:* carfilzomib (56 mg/m^2^) and dexamethasone*; QLQ-C30:* EORTC core Quality of Life Questionnaire; *QoL:* quality of life; Vd: bortezomib and dexamethasone; *W:* week.

A


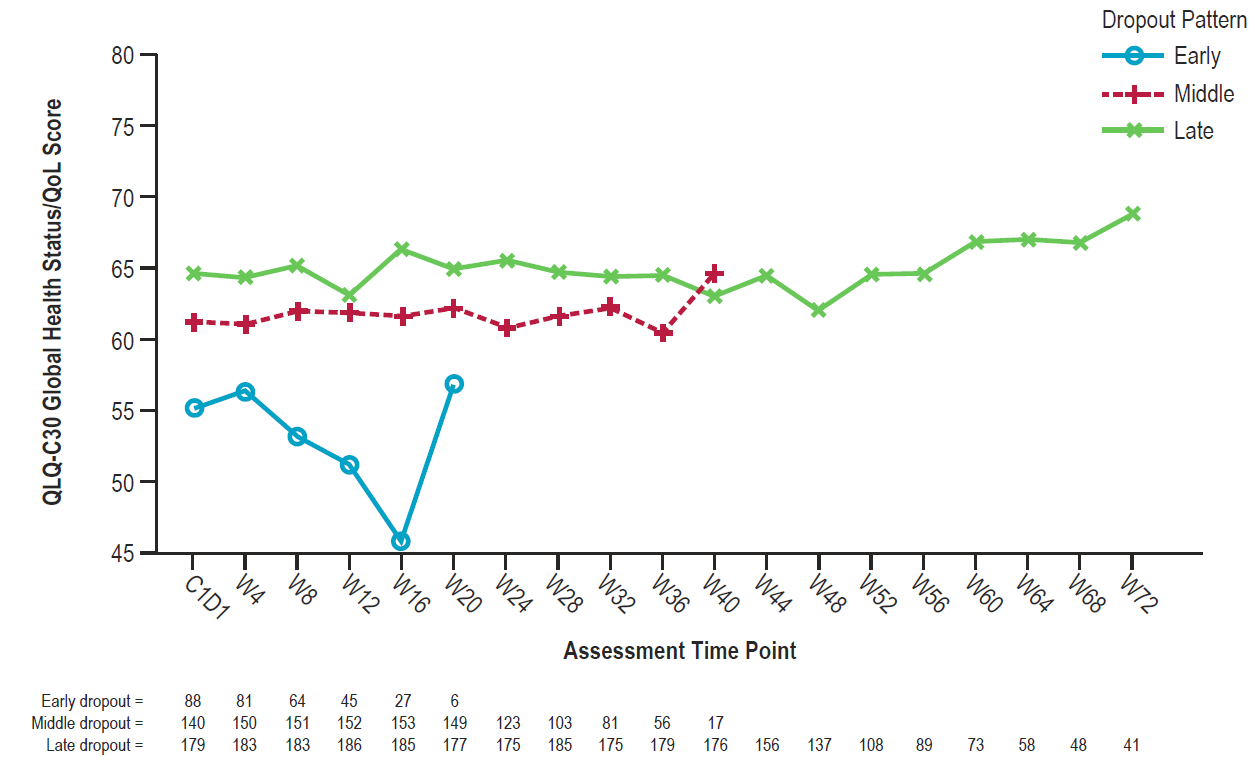


B


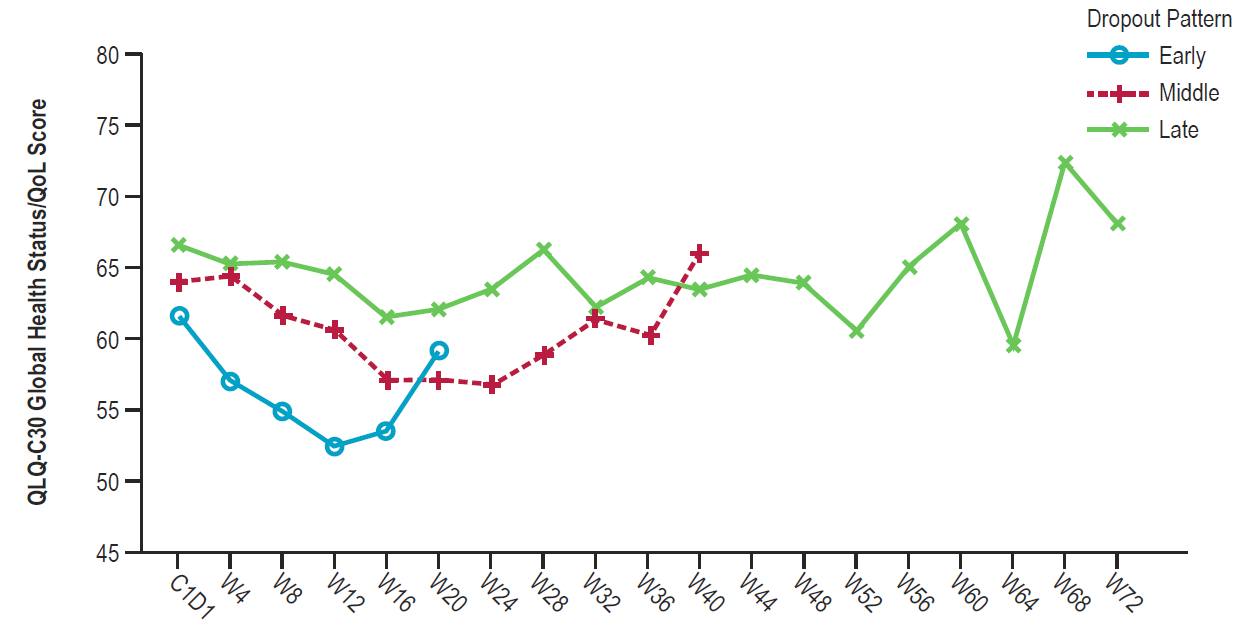


**Fig. S2 Mean estimates and standard errors by treatment group (for the subset of visits with assessments at day 1 of a cycle) in QLQ-C30 Global Health Status/QoL.** Overall *p* value for Kd56 versus Vd: <0.0001. *C1D1:* cycle 1 day 1; *HR-QoL:* health-related quality of life; *Kd56:* carfilzomib (56 mg/m^2^) and dexamethasone; *QLQ-C30:* Quality of Life Questionnaire-Core 30-item module; *QoL:* quality of life; *Vd:* bortezomib and dexamethasone; *W:* week.


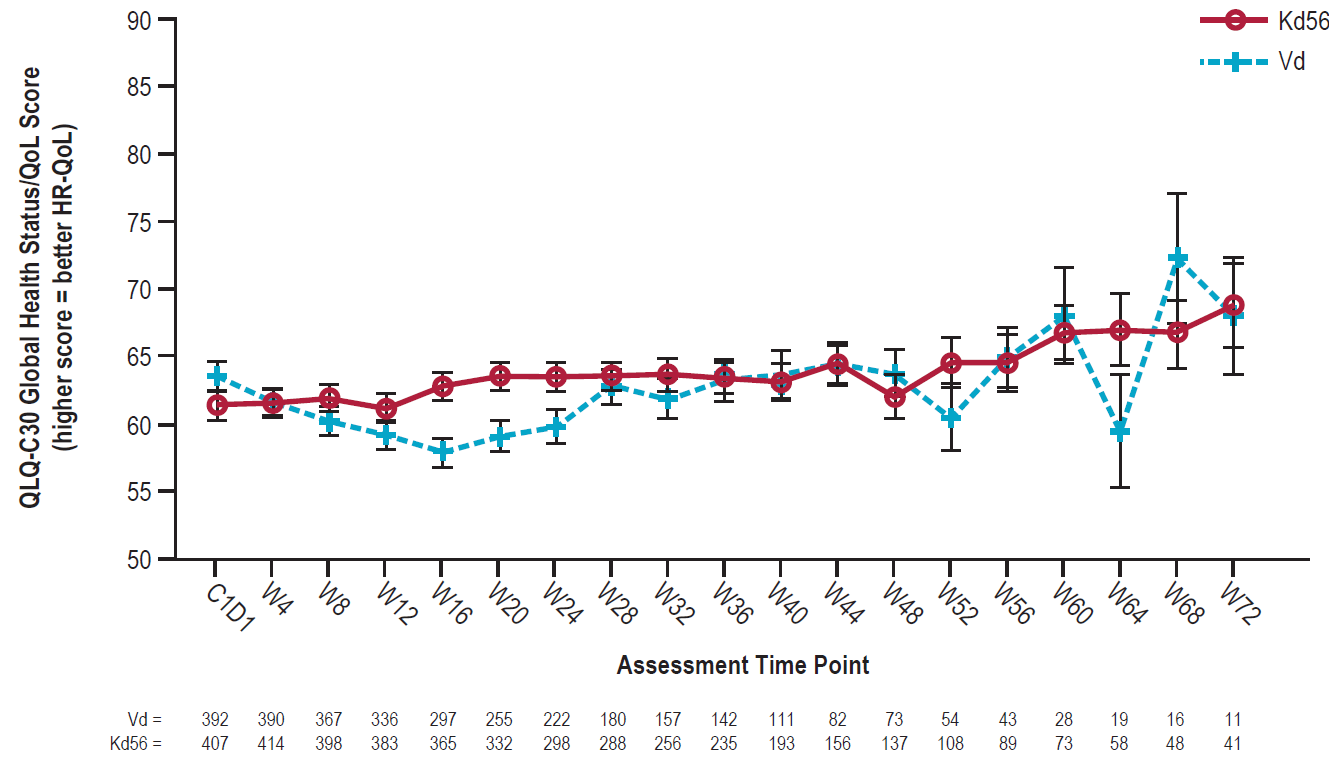

Supplement: Supplementary file 1 — Supplementary Information. [file 41408_2019_181_MOESM1_ESM.docx]
